# Echinatin mitigates sevoflurane-induced neurotoxicity through regulation of ferroptosis and iron homeostasis

**DOI:** 10.18632/aging.205622

**Published:** 2024-03-05

**Authors:** Yanqiu You, Xudong Zhou, Qiuqin Tang, Tianshou Zhao, Juan Wang, Hanqin Huang, Jibing Chen, Zhongquan Qi, Fujun Li

**Affiliations:** 1 Guangxi Key Laboratory of Special Biomedicine, School of Medicine, Guangxi University, Nanning 530004, China; 2 Ruikang Hospital Affiliated of Guangxi University of Chinese Medicine, Nanning 530011, China

**Keywords:** sevoflurane, Echinatin, ALOX12, neurotoxicity, ferroptosis

## Abstract

Surgery and anesthesia are vital medical interventions, but concerns over their potential cognitive side effects, particularly with the use of inhalational anesthetics like sevoflurane, have surfaced. This study delves into the neuroprotective potential of Echinatin against sevoflurane-induced neurotoxicity and the underlying mechanisms. Echinatin, a natural compound, has exhibited anti-inflammatory, antioxidant, and anticancer properties. Sevoflurane, while a popular anesthetic, is associated with perioperative neurocognitive disorders (PND) and neurotoxicity. Our investigation began with cellular models, where Echinatin demonstrated a significant reduction in sevoflurane-induced apoptosis. Mechanistically, we identified ferroptosis, a novel form of programmed cell death characterized by iron accumulation and lipid peroxidation, as a key player in sevoflurane-induced neuronal injury. Echinatin notably suppressed ferroptosis in sevoflurane-exposed cells, suggesting a pivotal role in neuroprotection. Expanding our research to a murine model, we observed perturbations in iron homeostasis, inflammatory cytokines, and antioxidants due to sevoflurane exposure. Echinatin treatment effectively restored iron balance, mitigated inflammation, and preserved antioxidant levels *in vivo*. Behavioral assessments using the Morris water maze further confirmed Echinatin’s neuroprotective potential, as it ameliorated sevoflurane-induced spatial learning and memory impairments. In conclusion, our study unveils Echinatin as a promising candidate for mitigating sevoflurane-induced neurotoxicity. Through the regulation of ferroptosis, iron homeostasis, and inflammation, Echinatin demonstrates significant neuroprotection both *in vitro* and *in vivo*. These findings illuminate the potential for Echinatin to enhance the safety of surgical procedures involving sevoflurane anesthesia, minimizing the risk of cognitive deficits and neurotoxicity.

## INTRODUCTION

Surgery and anesthesia are commonplace medical interventions worldwide, impacting millions of patients annually. Sevoflurane, a widely employed inhalational anesthetic in general surgery, is esteemed for its swift action and patient acceptance [1–3]. However, its use has been linked to a concerning issue known as perioperative neurocognitive disorders (PND). Despite its popularity, sevoflurane’s neurotoxic potential raises alarms, as it has been shown to hinder neurogenesis, induce neuronal apoptosis, and incite neuro-inflammatory responses [4]. Furthermore, sevoflurane’s detrimental impact extends to neural progenitor proliferation and neural stem cell self-renewal, often mediated by microglial neuro-inflammation [5, 6]. Nonetheless, the precise mechanisms underpinning sevoflurane-induced neuronal injury remain incompletely elucidated, impeding the development of effective neuroprotective strategies.

A relatively novel form of programmed cell death, ferroptosis, has garnered attention for its distinct features, characterized by excessive iron accumulation and lipid peroxidation, setting it apart from traditional cell death modalities like apoptosis, autophagy, pyroptosis, or necrosis [7]. Recent research has underscored the relevance of ferroptosis in central nervous system diseases, accentuating its role in neurodegeneration and cerebral injury [8, 9]. Several interconnected signaling pathways contribute to the initiation and execution of ferroptosis. Central to ferroptosis is the dysregulation of iron metabolism. Iron is taken up by cells via transferrin receptor 1 (TFR1) and divalent metal transporter 1 (DMT1). It is then stored in ferritin and released through ferroportin (FPN) [10, 11]. In ferroptosis, increased iron uptake and reduced iron export contribute to the accumulation of intracellular iron. Several genes and proteins are associated with the regulation of ferroptosis, including p53, NFE2 like bZIP transcription factor 2 (NRF2), and the lipid repair enzyme acyl-CoA synthetase long-chain family member 4 (ACSL4) [12, 13]. These factors influence iron metabolism, Reactive Oxygen Species (ROS) generation, and lipid peroxidation. GPX4 is an enzyme that detoxifies lipid hydroperoxides, preserving membrane integrity. In ferroptosis, GPX4 activity is inhibited, further exacerbating lipid peroxidation [14]. The mammalian lipoxygenase Arachidonate 12-lipoxygenase (ALOX12) has been implicated in the regulation of ferroptosis [15]. Understanding these intricate signaling pathways is crucial for developing potential therapeutic strategies to either promote or inhibit ferroptosis, depending on the context of various diseases and conditions.

Echinatin, a natural compound, possesses noteworthy medicinal properties and pharmacological activities, including anti-inflammatory and antioxidant effects [16, 17]. Moreover, Echinatin has shown promise in inhibiting the growth of certain cancer cells [17, 18]. Some studies suggest that Echinatin may have cardioprotective effects. It can help maintain cardiovascular health by reducing oxidative stress and inflammation [19]. Echinatin’s antioxidant and anti-inflammatory properties make it a potential candidate for protecting nerve cells from damage and degeneration. However, there is limited research assessing the role of Echinatin in diseases associated with nerve injury. This study aims to delve into the molecular mechanisms underpinning the neuroprotective potential of Echinatin against sevoflurane-induced neurotoxicity. By elucidating these mechanisms, we seek to contribute to the development of strategies to mitigate the adverse neurological effects associated with sevoflurane anesthesia.

## MATERIALS AND METHODS

### Cell culture and treatments

The immortalized mouse hippocampal cell line HT22, procured from CHI Scientific (Shanghai, China), was cultured in Dulbecco’s Modified Eagle’s Medium (DMEM) supplemented with 10% fetal bovine serum (Thermo Fisher Scientific, Waltham, MA, USA) and 1% penicillin-streptomycin (Thermo Fisher Scientific, Waltham, MA, USA). Cells were maintained at 37° C in a humidified atmosphere with 5% CO_2_. For sevoflurane treatment, HT22 cells were placed in a gas-tight chamber and exposed to 4% sevoflurane for 12 hours. Echinatin, obtained from Chengdu Alfa Biotechnology Co., Ltd. (Chengdu, China), was administered to cells at the specified concentrations for 24 hours. To inhibit ferroptosis, Ferrostain-1 (1 μM; Beyotime Co., Ltd., Beijing, China) was employed. Lentiviruses for ALOX12 overexpression/knockdown were constructed by Genechem Co., Ltd. (Nanjing, China).

### Western blot analysis

To conduct Western blot analysis, cells were lysed using radioimmunoprecipitation assay (RIPA) buffer (Beyotime Co., Ltd., Shanghai, China) containing proteinase and phosphatase inhibitors (Thermo Fisher Scientific, Waltham, MA, USA). Equal quantities of protein (20 μg) were loaded onto 8% sodium dodecyl sulfate-polyacrylamide gel (SDS-PAGE) and subsequently transferred to polyvinylidene fluoride (PVDF) membranes (Millipore, Billerica, MA, USA). Following protein transfer, the membranes were blocked using 5% non-fat milk and then incubated overnight at 4° C with primary antibodies. After three washes, the membranes were incubated for an additional 1 hour with horseradish peroxidase-conjugated secondary antibodies. The primary antibodies used were as follows: cleaved-caspase3 antibody (1:1000, ab32042, Abcam); Bcl-2 antibody (1:2000, ab182858, Abcam); Bax antibody (1:2000, ab32503, Abcam); GCL antibody (1:3000, ab190685, Abcam); NQO1 antibody (1:1500, ab80588, Abcam); Prx1 antibody (1:3000, ab106834, Abcam); FPN/ SLC40A1 (1:1000, ab239583, Abcam); TFR1 (1:2000, ab109259, Abcam); DMT1 (1:3000, ab55753, Abcam); p53 (1:1500, ab32049, Abcam); p21 (1:2000, ab109119, Abcam); SLC7A11 (1:2000, ab175186, Abcam); ALOX12 (1:1000, ab211506, Abcam); β-actin (1:6000, ab8226, Abcam). Secondary antibodies employed were: HRP-linked anti-mouse IgG (1:8000, 7076P2, Cell Signaling Technology, Inc.) HRP-linked anti-rabbit IgG (1:5000, 7074P2, Cell Signaling Technology, Inc.).

### Lactate dehydrogenase (LDH) release assay

LDH is an intracellular enzyme that is released into the supernatant upon cell death, serving as an indicator of cell membrane integrity. To assess LDH release in this study, we utilized the LDH Cytotoxicity Assay Kit (Best Bio Co., Ltd., Beijing, China), following the manufacturer’s protocol.

### Flow cytometric analysis

To assess ROS generation, we utilized the Reactive Oxygen Species Assay Kit (Beijing Solarbio Science and Technology Co., Ltd. Beijing, China). Cells were cultured for 24 hours at 37° C and subsequently incubated with 10 μM Dichlorodihydrofluorescein diacetate assay (DCFH-DA) for 15 minutes in darkness. The stained cells were then analyzed using a BD Bioscience flow cytometer. For apoptosis analysis, collected cells underwent dual-staining with Annexin V-APC and 7-AAD (KeyGEN Co. Ltd., Nanjing, China) for 30 minutes. After centrifugation at 600 x g for 3 minutes, cell apoptosis levels (including early and late apoptosis) were determined via flow cytometry.

### Cell viability assay

The MTT (3-(4,5-dimethylthiazol-2-yl)-2,5-diphenyltetrazolium bromide) assay kit (Beijing Solarbio Science and Technology Co., Ltd. Beijing, China) is used to assess cell viability. First, cells are seeded in a 96-well plate and treated if necessary. After incubation, MTT solution is added, and cells are incubated again. Viable cells convert MTT into purple formazan crystals. After removing excess MTT solution, DMSO is added to dissolve the crystals. The absorbance at 490 nm is measured using a microplate reader. In the Typan Blue Staining procedure, cells were initially resuspended in 1.0 mL of fresh growth medium. Subsequently, a 10 μL aliquot was extracted from each sample and subjected to cell counting via a hemacytometer. Staining was performed using 0.4% Trypan Blue (Beijing Solarbio Science and Technology Co., Ltd., Beijing, China). Cell death was quantified as a percentage of blue-positive (indicating dead) cells relative to the total cell population, which included both blue-positive and blue-negative cells.

### Enzyme-linked immunosorbent assay

ELISA was employed to analyze hippocampal tissues or cultured HT22 cells. Tissues and cells were homogenized while maintained on ice. Commercial kits (Beijing Solarbio Science and Technology Co., Ltd. Beijing, China) were used to assess the levels of malondialdehyde (MDA), glutathione (GSH), and superoxide dismutase (SOD) following the manufacturer’s guidelines. Additionally, the concentrations of IL-1β, IL-6, and TNF-α in the culture medium or hippocampal tissues were determined using commercial ELISA kits (Beyotime Co., Ltd., Beijing, China). Arachidonic acid serves as a substrate for ALOX12, wherein active ALOX12 catalyzes the conversion of arachidonic acid into 12-Hydroxyeicosatetraenoic acid (12-HETE). Specifically, active ALOX12 transforms 12(S)-hydroperoxy tetraenoic eicosatetraenoic acid [12(S)-HpETE] into 12(S)-HETE (12-HETE). The quantification of 12-HETE levels was conducted using a 12-HETE Enzyme-Linked Immunosorbent Assay (ELISA) kit obtained from Abcam, Cambridge, MA, USA.

### FerroOrange staining

For the assessment of intracellular Fe^2+^ levels, HT22 cells were initially plated in 6-well plates at a density of 1 × 10^6^ cells per well. Following the respective treatments, the HT22 cells were subjected to staining using serum-free phenol red-free DMEM (Thermo Fisher Scientific, Waltham, MA, USA) supplemented with 1 μM FerroOrange (Dojindo, Japan). This staining process occurred over a 30-minute duration at 37° C within a CO_2_ incubator. Subsequently, fluorescence measurements were performed using a fluorescence digital microscopy system (Olympus IX 53, Tokyo, Japan). The quantification of fluorescence intensity was accomplished through the utilization of ImageJ software (http://imagej.net/ImageJ).

### Network pharmacology

In the realm of network pharmacology, potential pharmacological targets associated with Echinatin were obtained through accessible online resources, including SwissTargetPrediction (http://swisstargetprediction.ch/) and TARGET PREDICTION (https://prediction.charite.de/subpages/target_prediction.php). The GeneCards database (https://www.genecards.org/) and OMIM database (https://omim.org/) were accessed to collect ferroptosis-related genes. Subsequently, candidate genes underwent correction and identification procedures using the UniProt database (https://www.uniprot.org/). The jvenn online software (https://jvenn.toulouse.inrae.fr/app/index.html) was utilized to identify common genes between the predicted target genes of Echinatin and those associated with ferroptosis. Furthermore, the Metascape database (http://metascape.org/) was employed to construct a protein–protein interaction (PPI) network, perform Gene Ontology (GO) enrichment analysis, and explore Kyoto Encyclopedia of Genes and Genomes (KEGG) pathway analyses.

### Animals

Fifteen male C57BL/6 mice, aged 6 to 8 weeks, were obtained from the Animal Experiment Center at Guangxi Medical University. The mice were housed in a controlled environment with a 12-hour light-dark cycle at 24 ± 2° C and 60 ± 10% humidity for 4 weeks. They had ad libitum access to food and water. All experimental procedures strictly adhered to the guidelines and regulations set forth by the Animal Care and Ethics Committee of Guangxi Medical University. The mice were divided into three groups: the control group, the sevoflurane group (Sevo), and the sevoflurane and Echinatin group (Sevo+Ech), with each group comprising 5 mice. Mice in the Sevo+Ech group were intraperitoneally administered Echinatin (20 mg/kg), while those in the Sevo group received an equivalent volume of normal saline, 2 hours before exposure to a mixture of 3% sevoflurane and 60% oxygen (balanced with nitrogen). A similar procedure was performed for the negative control group. To assess the learning and memory abilities of the mice, Morris water maze (MWM) tests were conducted following established protocols [20]. Briefly, one week prior to the initiation of sevoflurane, mice from each experimental group underwent training sessions for the Morris water maze task. After a 24-hour interval post sevoflurane anesthesia, the assessment was conducted. mice were placed in the water from four different starting points. The time taken to locate the hidden platform (escape latency) and the number of platform crossings were recorded. In a subsequent phase with the platform removed, the mice swam randomly from new starting points, and their total swimming distance was measured.

### Statistical analysis

The data were subjected to statistical analysis using SPSS 22.0 and GraphPad Prism 9.0 software. Results are presented as mean ± standard deviation. Student’s t-test was utilized for comparisons between two groups, whereas one-way analysis of variance (ANOVA) followed by Tukey’s post hoc analyses was employed for comparisons involving multiple groups. Statistical significance was defined as p<0.05.

### Data availability

The datasets used and/or analyzed during the current study are available from the corresponding author on reasonable request.

## RESULTS

### Echinatin mitigates sevoflurane-induced apoptosis in HT22 cells

**Figure 1:**
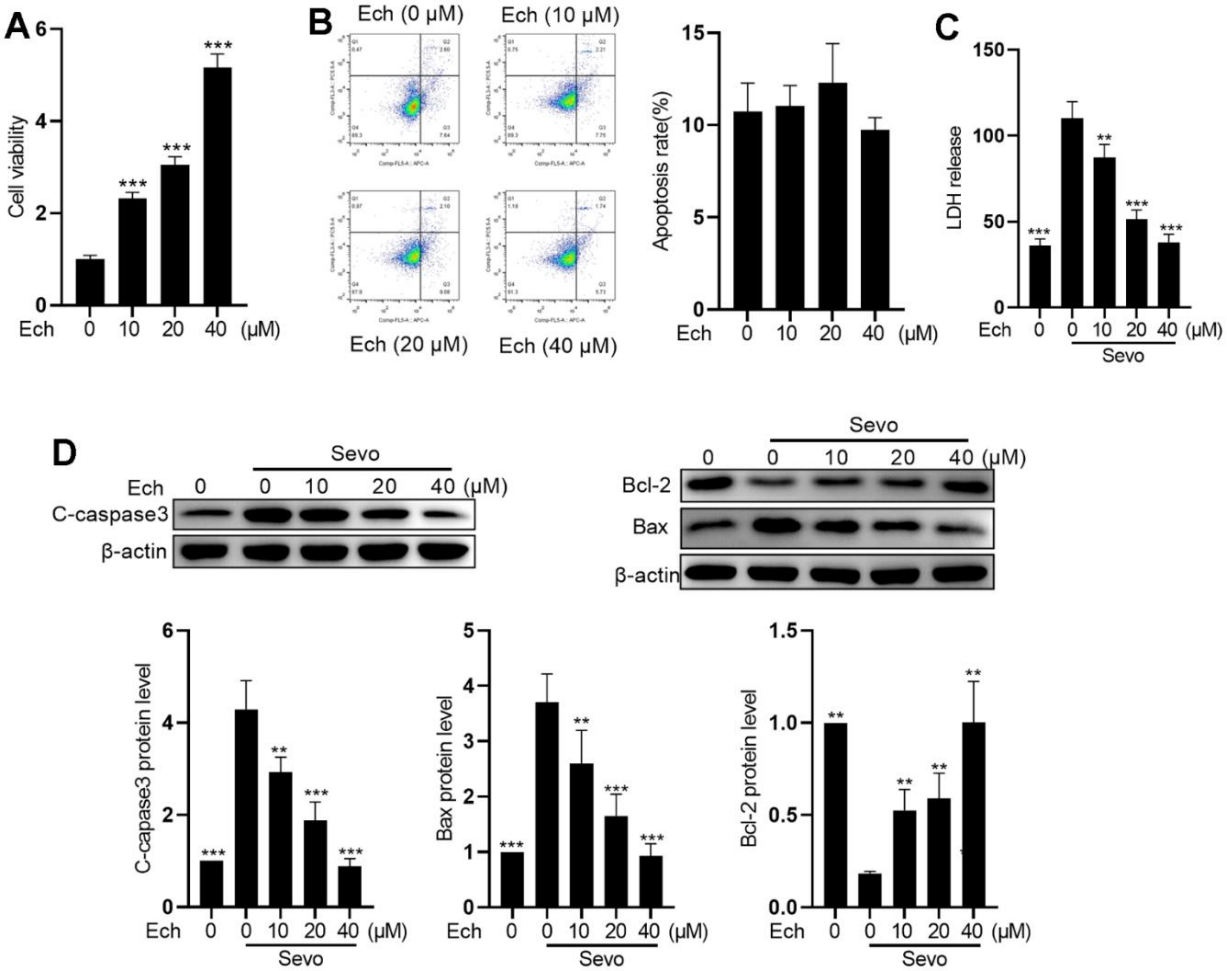
**Echinatin mitigates sevoflurane-induced apoptosis in HT22 cells.** HT22 cells were subjected to Echinatin treatment (0-40 μM) for a duration of 24 hours, followed by exposure to sevoflurane or control conditions. (**A**) Cell vitality was measured by MTT assay. (**B**) Cell apoptosis was measured by flow cytometry. (**C**) LDH release was measured by LDH assay kits. (**D**) Protein levels of cleaved-caspase3, Bcl-2, and Bax were measured by western blot. The data are presented as the mean ± SD. Ech, Echinatin; Sevo, sevoflurane. Compared with the Sevo+ Ech (0 μM) group, **P<0.01, ***P<0.001.

To explore the protective effect of Echinatin against sevoflurane-induced neurotoxicity, we initially assessed the cytotoxicity of Echinatin in HT22 cells. The results from the MTT cell viability assay revealed a dose-dependent enhancement of HT22 cell viability by Echinatin (Figure 1A). Flow cytometry analysis of apoptosis demonstrated that Echinatin exhibited no cytotoxicity at doses below 40 μM in HT22 cells (Figure 1B). Subsequently, we examined the influence of Echinatin on sevoflurane-induced apoptosis in HT22 cells. The release of cytoplasmic enzymes, such as LDH, into the supernatant following cell death serves as an indicator of cell membrane integrity. Our findings indicated that Echinatin significantly reduced the sevoflurane-induced LDH release (Figure 1C). Furthermore, we assessed apoptosis-associated proteins in sevoflurane-treated HT22 cells. As depicted in Figure 1D, Echinatin downregulated pro-apoptotic proteins Bax and cleaved-caspase3 while upregulating the anti-apoptotic protein Bcl-2 (Figure 1D). These results suggest that Echinatin has the potential to attenuate sevoflurane-induced apoptosis in HT22 cells.

### Echinatin alleviates oxidative stress and inflammation in sevoflurane-exposed HT22 cells

**Figure 2:**
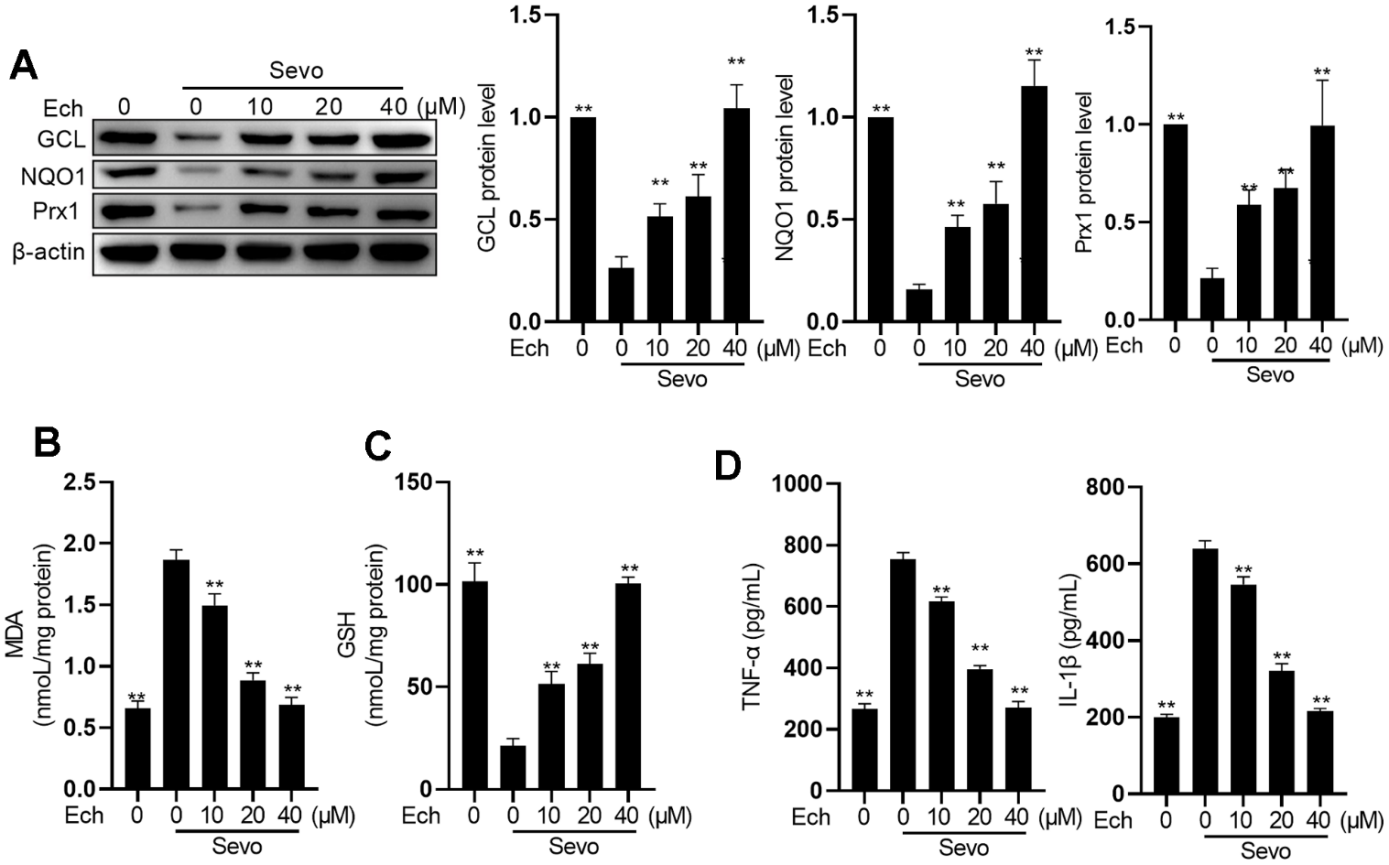
**Echinatin restrains sevoflurane-induced oxidative stress and inflammatory cytokines.** HT22 cells were subjected to Echinatin treatment (0-40 μM) for a duration of 24 hours, followed by exposure to sevoflurane or control conditions. (**A**) Protein levels of GCL, NQO1, and Prx1 were measured by western blot. (**B**–**D**) The levels of MDA, GSH, TNF-α and IL-1β were measured by commercial kits. The data are presented as the mean ± SD. Ech, Echinatin; Sevo, sevoflurane. Compared with the Sevo+ Ech (0 μM) group, **P<0.01, ***P<0.001.

To investigate the impact of Echinatin on oxidative stress and inflammation in HT22 cells exposed to sevoflurane, we exposed the cells to different concentrations of Echinatin (0, 10, 20, 40 μM) before administering sevoflurane. Sevoflurane treatment led to a reduction in the expression of anti-oxidative factors Heme oxygenase 1 (HO-1), NAD(P)H quinone dehydrogenase 1 (NQO1), Glutamate-cysteine ligase catalytic subunit (GCL), and Peroxiredoxin 1 (Prx1), which was counteracted by Echinatin (Figure 2A). In comparison to the control group, sevoflurane significantly increased MDA activity while decreasing GSH levels. Echinatin treatment, however, effectively suppressed MDA activity and concentration-dependently elevated GSH levels (Figure 2B, 2C). Moreover, Echinatin notably attenuated the sevoflurane-induced production of IL-1β and TNF-α in HT22 cells (Figure 2D). These findings collectively indicate that Echinatin inhibits sevoflurane-induced oxidative stress and inflammation in HT22 cells.

### Echinatin suppresses ferroptosis in sevoflurane-exposed HT22 cells

**Figure 3:**
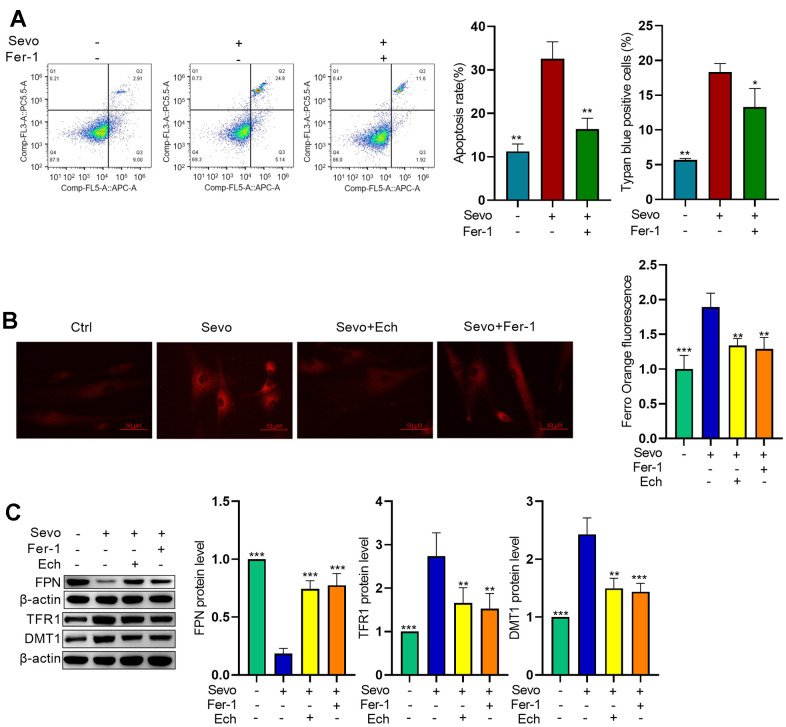
**Echinatin inhibits sevoflurane-induced ferroptosis in HT22 cells.** HT22 cells were subjected to Fer-1 (1 μM) treatment or Echinatin (40μM) treatment for 24 hours, followed by exposure to sevoflurane or control conditions. (**A**) Cell apoptosis was measured by flow cytometry. (**B**) Intracellular Fe^2+^ detected by FerroOrange. (**C**) Protein levels of FPN, TFR1, and DMT1 were measured by western blot. The data are presented as the mean ± SD. Compared with the Sevo group, *P<0.05, **P<0.01.

Targeting ferroptosis is considered an effective strategy for mitigating sevoflurane-induced brain injury. Therefore, we investigated the impact of Echinatin on ferroptosis. To validate whether ferroptosis is involved in sevoflurane-induced apoptosis, we employed Fer-1, a ferroptosis inhibitor. Flow cytometric assays and trypan blue exclusion staining results demonstrated that inhibiting ferroptosis reduced sevoflurane-induced apoptosis (Figure 3A). One hallmark of ferroptosis is the accumulation of iron. To assess iron homeostasis in our model, we measured iron deposition levels using FerroOrange staining. The results revealed an increase in ferrous ions in sevoflurane-treated cells, while Echinatin and Fer-1 decreased cellular Fe^2+^ content compared to that in sevoflurane-treated cells (Figure 3B). We also examined the expression of iron homeostasis-related proteins via western blot. The results showed higher protein levels of TFR1 and DMT1 in HT22 cells in the sevoflurane group compared to the control group. Conversely, FPN protein levels were lower. However, these alterations were reversed by both Echinatin and Fer-1 (Figure 3C). These findings suggest that Echinatin may alleviate sevoflurane-induced apoptosis by inhibiting ferroptosis.

### Key targets and enriched pathways common to Echinatin and ferroptosis

**Figure 4:**
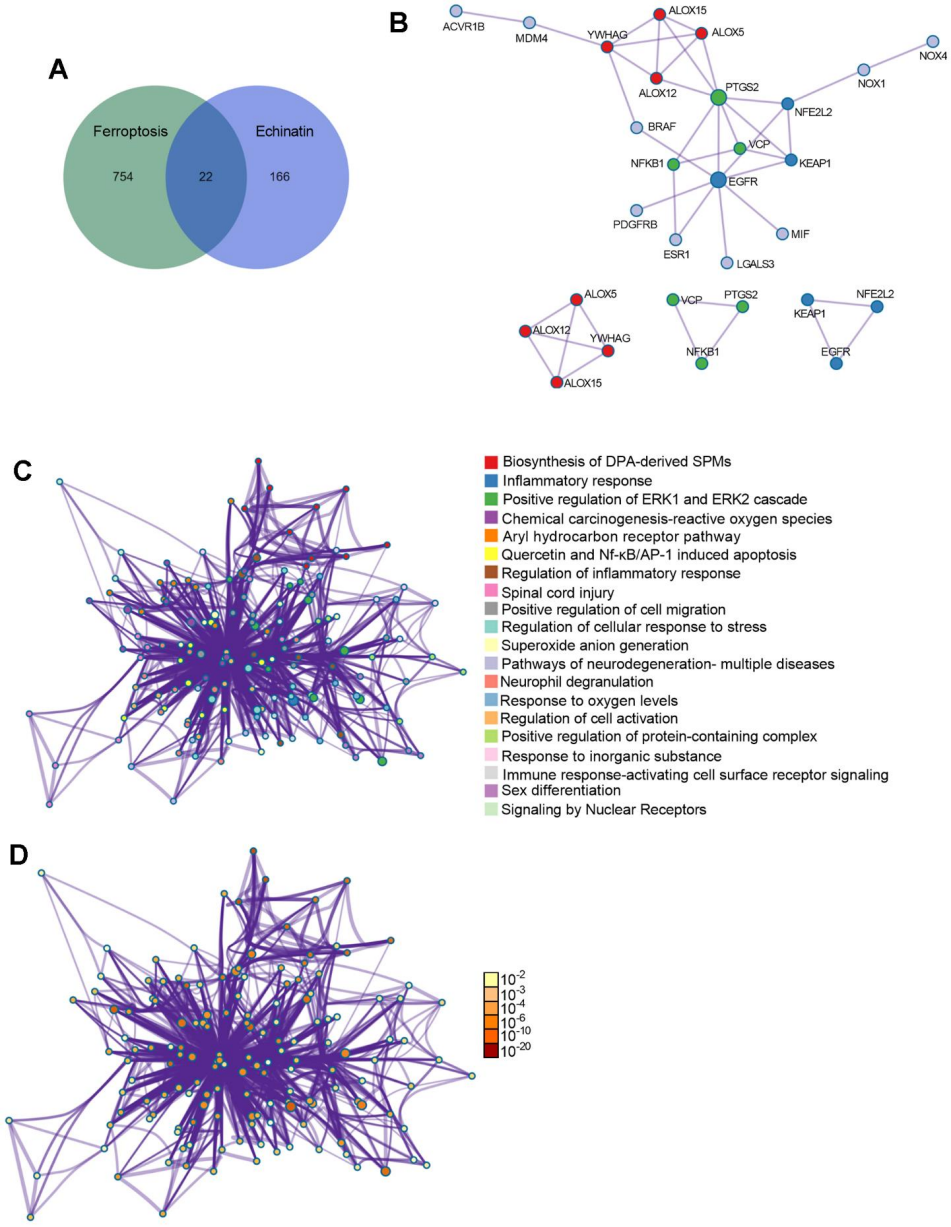
**The co-differentially expressed genes in Echinatin and ferroptosis.** (**A**) The Venn graph of overlap genes in both Echinatin and ferroptosis. (**B**) PPI network and key functional modules of Echinatin against ferroptosis. The enrichment of overlap genes using the Metascape database. Network of enriched terms: (**C**) colored by cluster ID, where nodes that share the same cluster ID are typically close to each other; (**D**) colored by p-value, where terms containing more genes tend to have a more significant p-value.

To gain deeper insights into the function of Echinatin, we performed an analysis to identify potential pharmacological targets of Echinatin using the online tool SwissTargetPrediction and TARGET PREDICTION. This analysis yielded 188 targets associated with Echinatin. Additionally, we obtained 767 ferroptosis-related genes from the OMIM and Genecards databases. In order to uncover shared regulatory elements, we identified 22 genes that were common to both Echinatin and ferroptosis (Figure 4A). To further explore the significance of these genes, we constructed a Protein-Protein Interaction (PPI) network using “Metascape” and identified three biological functional modules through “Molecular Complex Detection (MCODE)” (Figure 4B). Subsequent Gene Ontology (GO) and Kyoto Encyclopedia of Genes and Genomes (KEGG) enrichment analyses revealed that these overlapping genes were associated with processes such as inflammatory response, oxygen regulation, regulation of ERK1 and ERK2 cascade, regulation of cellular response to stress, Nf-κB induced apoptosis, and more (Figure 4C, 4D). Of particular interest among these common genes is ALOX12, as it is known to play a crucial role in p53-mediated ferroptosis [15]. Therefore, we proceeded to investigate the relationship between ALOX12 and Echinatin.

### Echinatin suppresses sevoflurane-induced ferroptosis by inhibiting ALOX12 activity

**Figure 5:**
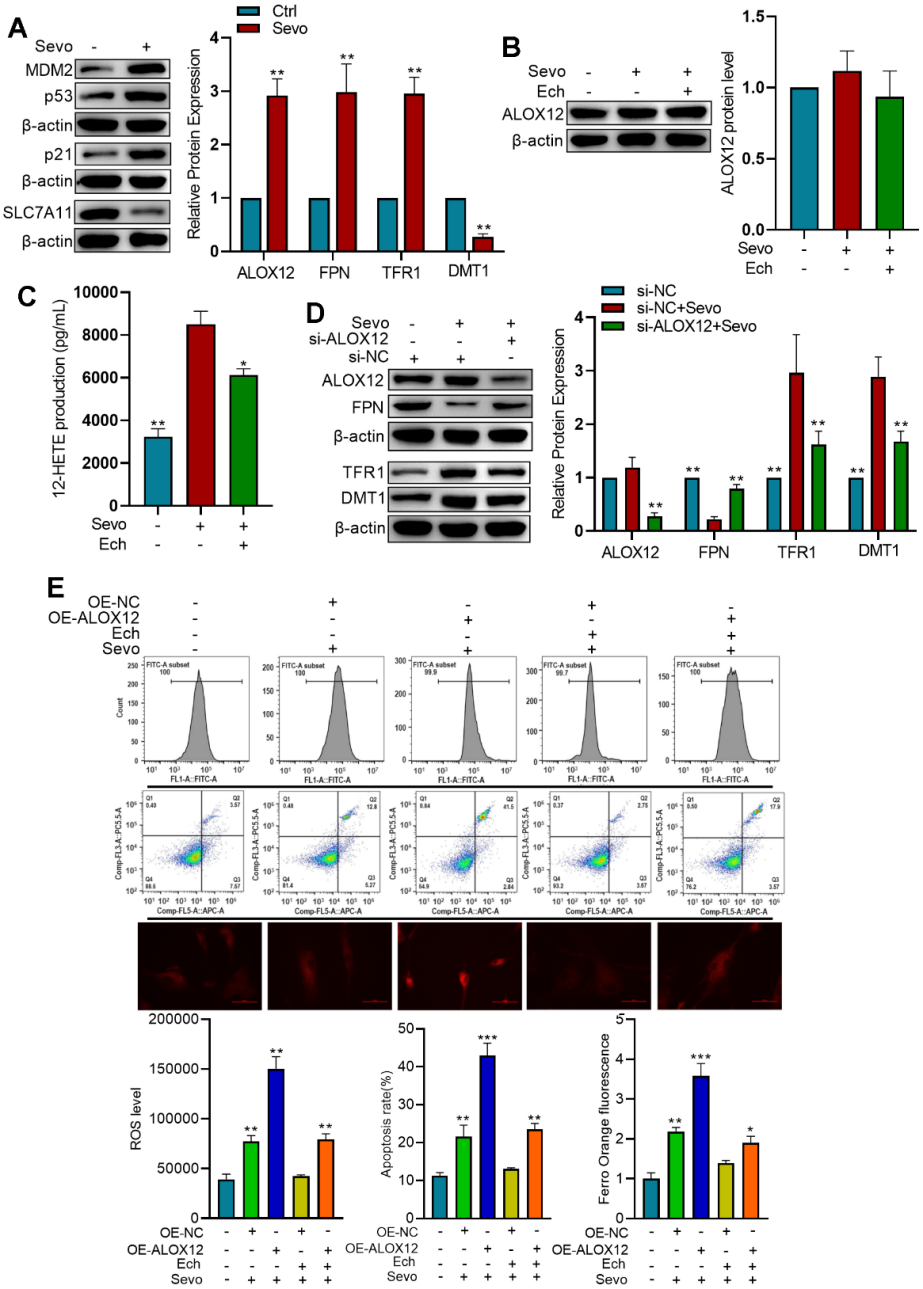
**Echinatin inhibits sevoflurane-induced ferroptosis via ALOX12.** (**A**) HT22 cells were exposed to either sevoflurane or control conditions. Protein levels of p53, p21, and SLC7A11 were measured by western blot. Compared with the Ctrl group, **P<0.01. (**B**) HT22 cells were subjected to Echinatin treatment (40 μM) for a duration of 24 hours, followed by exposure to sevoflurane or control conditions. Protein level of ALOX12 was measured by western blot. (**C**) ALOX12 activity was measured by detecting 12-HETE levels by ELISA. Compared with the Sevo group, *P<0.05, **P<0.01. (**D**) HT22 cells infected with shRNA-control (si-NC) lentivirus or shRNA-ALOX12 (si-ALOX12) lentivirus, followed by exposure to sevoflurane or control conditions. Protein levels of ALOX12, FPN, TFR1, and DMT1 were measured by western blot. Compared with the Sevo+si-NC group, *P<0.05, **P<0.01. (**E**) HT22 cells were infected with lentiviruses carrying either the overexpression control (OE-NC) or ALOX12 overexpression (OE-ALOX12) constructs, and subsequently subjected to Echinatin treatment (40 μM) followed by exposure to sevoflurane. Cell apoptosis and ROS levels were measured using flow cytometry. Intracellular Fe^2+^ detected by FerroOrange. Compared with indicated group, **P<0.01, ***P<0.001. The data are presented as the mean ± SD. Ech, Echinatin; Sevo, sevoflurane.

In HT22 cells exposed to sevoflurane, we observed increased expression of MDM2, p53, and p21, along with decreased expression of SLC7A11. These findings suggest that sevoflurane induces ferroptosis (Figure 5A). Western blot analysis revealed that while sevoflurane and Echinatin did not alter the protein levels of ALOX12, sevoflurane treatment enhanced the lipoxygenase activity of ALOX12, an effect that was mitigated by Echinatin (Figure 5B, 5C). Moreover, knockdown of ALOX12 expression using lentivirus-mediated shRNAs resulted in reduced TFR1 and DMT1 expressions, as well as increased FPN expression. This indicates that sevoflurane-induced ferroptosis is dependent on ALOX12 (Figure 5D). Upon sevoflurane treatment, overexpression of ALOX12 increased ROS levels, cell apoptosis, and Fe^2+^ content in HT22 cells. However, Echinatin effectively counteracted the effects of ALOX12 overexpression, underscoring that Echinatin inhibits sevoflurane-induced ferroptosis through the regulation of ALOX12 (Figure 5E).

### Echinatin mitigates sevoflurane-induced learning and memory impairments

**Figure 6:**
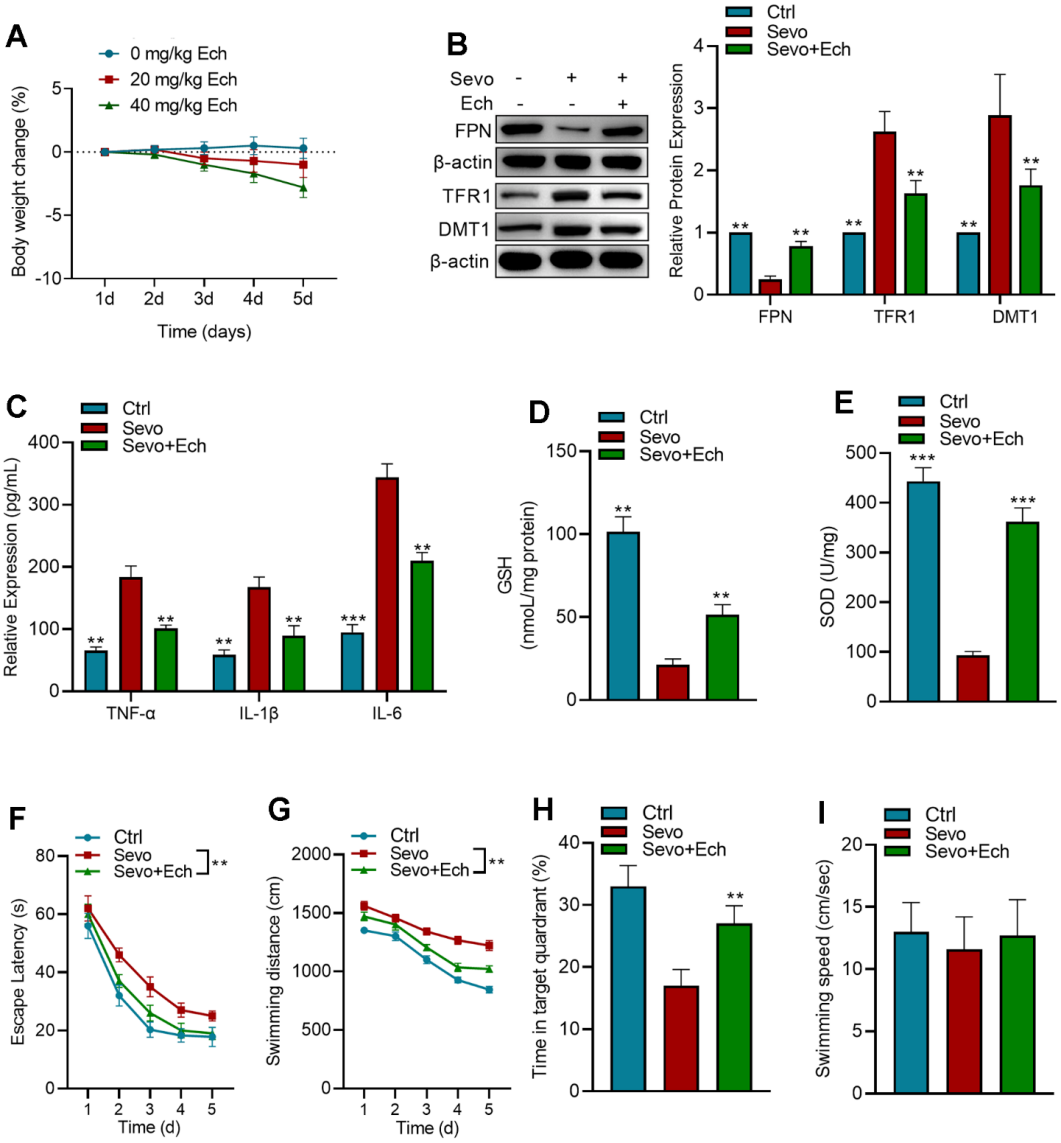
**Echinatin enhances learning and memory capabilities of mice exposed to sevoflurane.** (**A**) Changes in mice body weight after injection of different concentrations of Echinatin. For *in vivo* experiments, mice exposed to sevoflurane (3%) were intraperitoneally administered Echinatin at a dose of 20 mg/kg. (**B**) Protein levels of FPN, TFR1, and DMT1 in hippocampal tissue were assessed using western blot analysis. (**C**–**E**) Expression levels of TNF-α, IL-1β, IL-6, GSH, and SOD in hippocampal tissue were measured by commercial kits. (**F**) Escape latency during Morris water maze testing. (**G**) Swimming distance during Morris water maze testing. (**H**) Time in target quadrant during Morris water maze testing. (**I**) Swimming velocity during Morris water maze testing. The data are presented as the mean ± SD. Ech, Echinatin; Sevo, sevoflurane. Compared with the Sevo group, **P<0.01, ***P<0.001.

In order to evaluate whether different concentrations of Echinatin were toxic to mice, we observed the changes in body weight of mice treated with 0, 20mg/kg and 40mg/kg Echinatin, and the results showed (Figure 6A) that 20mg/kg Echinatin had no significant effect on body weight of mice, and there were no deaths during the experiment. To assess the impact of Echinatin on sevoflurane-induced memory deficits and ferroptosis, we evaluated the expression of iron homeostasis-related proteins in mice and conducted behavioral assessments using the Morris water maze. We observed that Echinatin significantly counteracted the effects of sevoflurane on the expression of FPN, TFR1, and DMT1 in mice (Figure 6B). Moreover, Echinatin effectively reduced sevoflurane-induced elevation of inflammatory factors TNF-α, IL-1β, and IL-6 in the hippocampus (Figure 6C). Additionally, Echinatin restored the levels of SOD and GSH in the hippocampus of mice (Figure 6D, 6E). In the Morris water maze test, there were no discernible distinctions in the swimming velocities among the different groups. Sevoflurane-treated mice exhibited significantly longer escape latency, increased relative swimming distance, and spent less time exploring the target quadrant compared to control mice. However, Echinatin treatment led to a significant reduction in escape latency and swimming distance, along with an increase in the frequency of target quadrant crossings (Figure 6F–6I). In summary, these results demonstrate that Echinatin alleviates memory deficits induced by sevoflurane by inhibiting ferroptosis.

## DISCUSSION

Our study delved into the intricate relationship between ferroptosis and sevoflurane-induced neurotoxicity, shedding light on the potential of Echinatin as a neuroprotective agent. Ferroptosis, characterized by iron dysregulation and lipid peroxidation, emerges as a critical player in the pathogenesis of anesthesia-induced neurotoxicity [21]. Our data showed that Echinatin, a natural compound known for its anti-inflammatory and antioxidant properties, demonstrates promising neuroprotective effects both *in vitro* and *in vivo*.

Oxidative stress and inflammation are key players in the pathogenesis of anesthesia-induced neurotoxicity. Oxidative stress occurs when ROS accumulate as a result of an imbalance between the production of ROS and the activity of the cellular antioxidant system. The correct spatiotemporal control of ROS production and activity is crucial to many physiological functions, including neuronal fate and development [22]. However, excessive ROS levels result in damage to DNA, RNA, proteins, mitochondria, and lipids [23]. Our results revealed that Echinatin effectively countered the sevoflurane-induced reduction in anti-oxidative factors such as HO-1, NQO1, GCL, and Prx1. These factors play crucial roles in the cellular defense against oxidative stress, and their restoration by Echinatin suggests a potential mechanism of neuroprotection. Additionally, Echinatin appeared to regulate key markers of oxidative stress, with a suppression of MDA activity and an elevation of levels. MDA is a marker of lipid peroxidation and oxidative damage, while GSH is a critical antioxidant involved in detoxifying ROS [24, 25]. The ability of Echinatin to modulate these factors highlights its potential in combating oxidative stress in neuronal cells. Inflammation is another facet of anesthesia-induced neurotoxicity that we examined in our study. In response to sevoflurane exposure, HT22 cells exhibited increased production of pro-inflammatory cytokines IL-1β and TNF-α. However, Echinatin treatment notably suppressed the production of these cytokines, suggesting its potential in dampening neuro-inflammation. Given the well-established links between inflammation and neuronal damage, this anti-inflammatory property of Echinatin is of particular interest.

Considering the intricate relationship between ferroptosis and neuronal damage, we delved into the possibility that Echinatin may influence ferroptosis in sevoflurane-exposed neuronal cells. To explore this, we employed Fer-1, a ferroptosis inhibitor, and observed that inhibiting ferroptosis effectively reduced sevoflurane-induced apoptosis. This suggests a potential overlap between ferroptosis and apoptosis in our experimental model. While our research findings indeed demonstrate the inhibitory effect of Echinatin on cellular apoptosis, it is worth noting that in alternative investigations, Echinatin has exhibited the capacity to enhance apoptosis in cancer cells [18, 26, 27]. Iron, as a central player in ferroptosis [28], was a focus of our investigation. We found that Echinatin treatment reduced cellular Fe^2+^ content in HT22 cells exposed to sevoflurane, indicating a potential regulation of iron homeostasis.

Our investigation also ventured into the realm of potential pharmacological targets for Echinatin. Subsequent analysis of these genes led to the identification of biological functional modules associated with processes such as inflammatory response, oxygen regulation, cellular stress response, Nf-κB induced apoptosis, and more. Among these common genes, ALOX12, a lipoxygenase with implications in p53-mediated ferroptosis, emerged as a key player in our study.

While p53 has long been recognized for its pivotal role in imposing cell-cycle arrest, senescence, and apoptosis as essential barriers to cancer development, emerging evidence has illuminated an additional facet of its activity - metabolic regulation, which profoundly contributes to tumor suppression [29]. Notably, p53 has been implicated in the modulation of ferroptotic responses through its influence on various metabolic targets [30–32]. Among these, ALOX12, although seemingly dispensable for the overall development of mice [33], emerges as a critical player in p53-mediated ferroptosis. Intriguingly, p53 can indirectly activate ALOX12 function through the transcriptional repression of SLC7A11, a pivotal component of the cystine/glutamate antiporter system, thereby priming cells for ALOX12-dependent ferroptosis when confronted with heightened levels of ROS stress [15]. Oxidative stress, implicated in a myriad of pathological processes, extends its malevolent influence to neurodegeneration in the context of post-traumatic stress disorder (PTSD). In this intricate landscape, ALOX12 takes center stage. Notably, specific ALOX12 variants, such as rs1042357 and rs10852889, have been associated with a reduction in the thickness of the right prefrontal cortex in patients afflicted with PTSD [34]. Furthermore, the interplay between pro-inflammatory cytokines, such as TNFα and IL-1β, and ALOX12 further underscores the complexity of ALOX12’s role. These cytokines have been shown to stimulate increased ALOX12 expression within islet cells, leading to the production of 12-HETE. This, in turn, serves as a trigger for heightened expression of Nox1 in islet cells, culminating in an amplification of ROS levels within these cells [35]. Collectively, these lines of evidence highlight the intimate involvement of ALOX12 in cellular damage induced by oxidative stress. Although we did not observe significant changes in ALOX12 protein levels in response to sevoflurane exposure or Echinatin treatment, we did note alterations in ALOX12 activity. To confirm the impact of ALOX12 and Echinatin on ferroptosis, we examined cellular ROS levels, cell apoptosis, and Fe^2+^ content in HT22 cells exposed to sevoflurane. Overexpression of ALOX12 exacerbated ferroptosis-related features, while Echinatin effectively counteracted these effects. This finding highlights the potential of Echinatin in modulating ferroptosis by targeting ALOX12. In the murine model, conspicuous shifts in iron-related proteins were discerned, signifying perturbations in iron homeostasis consequent to sevoflurane exposure. Significantly, the administration of Echinatin ameliorated these perturbations, intimating its prospective prowess in orchestrating *in vivo* iron equilibrium. Furthermore, Echinatin counteracted sevoflurane-induced perturbations in inflammatory cytokines and antioxidants. Notably, behavioral assessments unequivocally demonstrated the marked amelioration in these behaviors following Echinatin treatment, aligning with the neuroprotection observed in our cellular and biochemical analyses.

In conclusion, our study provides valuable insights into the complex molecular mechanisms underlying anesthesia-induced neurotoxicity and highlights the potential for therapeutic interventions targeting ferroptosis. By elucidating the role of Echinatin in mitigating neurotoxicity, we contribute to the development of strategies aimed at reducing the adverse neurological effects associated with sevoflurane anesthesia. Further research into the precise mechanisms of Echinatin’s action and its clinical applicability holds great promise in enhancing the safety and outcomes of surgical procedures involving anesthesia.
